# Enhancing safety with an AI-empowered assessment and monitoring system for BSL-3 facilities

**DOI:** 10.1016/j.heliyon.2024.e40855

**Published:** 2024-12-16

**Authors:** Yi-Ling Fan, Ching-Han Hsu, Ju-Yu Wu, Ying-Ying Tsai, Wei J. Chen, Min-Shi Lee, Fang-Rong Hsu, Lun-De Liao

**Affiliations:** a Institute of Biomedical Engineering and Nanomedicine, National Health Research Institutes, 35, Keyan Road, Zhunan Town, Miaoli County, 350, Taiwan; b Department of Biomedical Engineering & Environmental Sciences, National Tsing-Hua University, Hsinchu, Taiwan; c Doctoral Program in Tissue Engineering and Regenerative Medicine, National Chung Hsing University, Taichung, Taiwan; d Center for Neuropsychiatric Research, National Health Research Institutes, 35, Keyan Road, Zhunan Town, Miaoli County, 350, Taiwan; e National Institute of Infectious Diseases and Vaccinology, National Health Research Institutes, 35, Keyan Road, Zhunan Town, Miaoli County, 350, Taiwan; f Department of Information Engineering and Computer Science, Feng Chia University, Taichung, 407, Taiwan

**Keywords:** Bioengineering, Biomedical instrumentation, Biomedical laboratory safety, Biosafety level-3 laboratory

## Abstract

**Introduction:**

The COVID-19 pandemic has created an urgent demand for research, which has spurred the development of enhanced biosafety protocols in biosafety level (BSL)-3 laboratories to safeguard against the risks associated with handling highly contagious pathogens. Laboratory management failures can pose significant hazards.

**Methods:**

An external system captured images of personnel entering a laboratory, which were then analyzed by an AI-based system to verify their compliance with personal protective equipment (PPE) regulations, thereby introducing an additional layer of protection. A deep learning model was trained to detect the presence of essential PPE items, such as clothing, masks, hoods, double-layer gloves, shoe covers, and respirators, ensuring adherence to World Health Organization (WHO) standards. The internal laboratory management system used a deep learning model to delineate alert zones and monitor compliance with the imposed safety protocols.

**Results:**

The external detection system was trained on a dataset consisting of 4112 images divided into 15 PPE compliance classes. The model achieved an accuracy of 97.52 % and a recall of 97.03 %. The identification results were presented in real time via a visual interface and simultaneously stored on the administrator's dashboard for future reference. We trained the internal management system on 3347 images, achieving 90 % accuracy and 85 % recall. The results were transmitted in JSON format to the internal monitoring system, which triggered alerts in response to violations of safe practices or alert zones. Real-time notifications were sent to the administrators when the safety thresholds were met.

**Conclusion:**

The BSL-3 laboratory monitoring system significantly reduces the risk of exposure to pathogens for personnel during laboratory operations. By ensuring the correct use of PPE and enhancing adherence to the imposed safety protocols, this system contributes to maintaining the integrity of BSL-3 facilities and mitigates the risk of personnel becoming infection vectors.

## 1 Introduction

The COVID-19 pandemic has highlighted the critical need for research on infectious pathogens. The U.S. Centers for Disease Control and Prevention (CDC) has established four biosafety levels (BSLs) [1]. Each level represents the degree of hazard posed by a pathogen, with higher levels corresponding to stricter laboratory regulations (see Table 1). BSL-1 laboratories handle pathogens that pose no disease risks to healthy adults with intact immune systems [2]. BSL-2 laboratories manage pathogens that may cause moderate harm to personnel and the environment [3], such as type-A influenza and Salmonella. These pathogens typically cause mild human illnesses. BSL-3 laboratories are designed for work involving microorganisms belonging to the third-tier risk group or second-tier microorganisms handled at high concentrations or in large-scale processes, particularly those with high aerosol dispersion risk levels [2]. Pathogens handled in these labs, including COVID-19, tuberculosis, yellow fever virus, and SARS-CoV, have the potential to cause severe or even fatal diseases. Personnel working in BSL-3 laboratories must undergo specialized training in terms of handling lethal pathogens. All experiments take place in ventilated cabinets, and personnel must wear protective clothing before entering these cabinets [4]. BSL-4 laboratories, representing the highest tier, are responsible for managing the most dangerous or unknown pathogens, which are capable of large-scale transmission through aerosols. The pathogens handled at this level, such as the smallpox and Ebola viruses, currently have no available vaccines or treatments. Personnel working in these laboratories are required to wear positive pressure protective suits with independent oxygen supplies [5]. A detailed summary of the various biosafety levels is provided in Table 1.

**Table 1:**
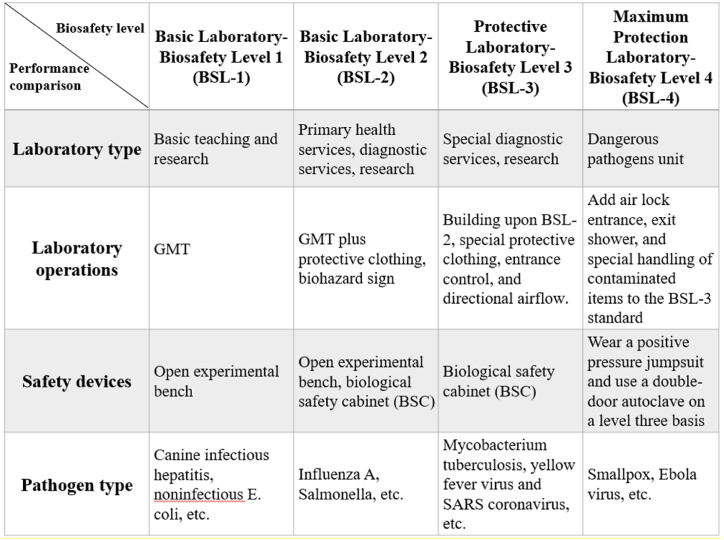
**According to their containment levels, biosafety laboratories are divided into four categories: P1, P2, BSL-3, and P4.** BSL classifications are based on the operational protocols, barriers, safety equipment, and facilities utilized by these laboratories. The four levels are as follows. BSL-1 (P1): laboratories conducting research on viruses and bacteria that cause no human diseases. BSL-2 (P2): laboratories conducting research on viruses and bacteria that may cause diseases in humans. BSL-3 (BSL-3): laboratories conducting research on viruses and bacteria that can cause severe or lethal diseases in humans. BSL-4 (P4): laboratories conducting research on viruses and bacteria that can cause severe or lethal human diseases for which no vaccines or treatment methods are available.

The COVID-19 outbreak has posed an unprecedented threat to humanity, creating an urgent need for global scientific research. Initially, studies were conducted in BSL-4 laboratories, such as P4 laboratories, due to the novel nature of the virus [5]. However, as scientists gained a better understanding of the virus and developed vaccines, research shifted to BSL-3 laboratories [6]. According to 2019 statistics from the Ministry of Health and Welfare of Taiwan, 18 institutions across the country are equipped with a total of 22 BSL-3 laboratories. Notably, 12 of these facilities were established or repurposed specifically for COVID-19 research over the past two years. Improper management or negligence that leads to the escape of pathogens can result in societal disruption, with severe consequences for both the economy and healthcare systems. Records of laboratory virus leaks worldwide have demonstrated their significant and harmful impacts [7,8]. A review of the laboratory-acquired infections (LAIs) occurring between 2011 and 2020 identified 338 cases; 26 LAIs were attributed to various pathogens, whereas 34 had unknown causes [9]. These findings underscore the critical need for effective training and monitoring schemes that can prevent LAIs [9].

The precautions undertaken when entering and exiting a laboratory are guided by the "Laboratory Safety Regulations for Biosafety Levels 1 to 3″ issued by the Taiwan Centers for Disease Control, which largely adhere to the guidelines set forth in the "Biosafety in Microbiological and Biomedical Laboratories (BMBL)" regulations of the CDC [10]. Personnel entering and exiting BSL-3 laboratories must comply with specific safety measures. For tasks involving infection risks, working alone in the laboratory is strongly discouraged. If only one person is present, another individual must observe their activities from outside the laboratory. Upon leaving the laboratory, personnel must record their departure times, and they must remove their protective equipment according to the prescribed procedures. The correct sequence for donning and doffing protective gear is posted at the laboratory entrance in accordance with the imposed regulations.

The standard procedure for entering a BSL-3 laboratory typically begins with individuals entering a preparation room. After completing the necessary procedures, they proceed to the anteroom, where they are required to equip a face mask and a respirator. Finally, two laboratory personnel verify each other's protective gear before entering the laboratory. However, this method of inspection and supervision is a time-consuming and labor-intensive approach, which can cause the process to become purely procedural [11]. In this study, the manual confirmation step is replaced with artificial intelligence (AI) recognition technology. Each person stands in front of an RGB camera, a photograph is taken, and the AI system quickly evaluates the image. If the assessment returns a result of "Allowed to enter," the person may proceed into the BSL-3 laboratory. If the system detects any issues, a second photograph and an AI evaluation are required before entry is permitted. The optimization and strict implementation of these procedures enhance both the safety and operational efficiency of the BSL-3 laboratory. Moreover, integrating AI recognition technology improves the ability to monitor the physical conditions of personnel [12,13], further reinforcing safety during laboratory operations. These technological advancements not only streamline operations but also help protect the health and safety of personnel. To date, no studies have investigated the use of AI technology for enhancing the safety and security of BSL-3 laboratories.

The objectives of this study are twofold: 1) to use an AI recognition technique to assess the proper use of protective clothing by laboratory personnel upon entering a BSL-3 laboratory and 2) to implement a monitoring system that can oversee the activities conducted by personnel within the laboratory. Traditionally, two people enter a BSL-3 laboratory together to verify each other's protective clothing. However, despite the presence of clear standards, individual experience variations and subjective opinions can lead to inconsistent judgments, resulting in potential oversight. To address this issue, AI recognition is introduced as an additional inspection step after personnel don their protective equipment, replacing the manual confirmation process. By using objective and standardized criteria, the system aims to reduce the risk of personnel being exposed to pathogens while conducting experiments in the BSL-3 laboratory. For internal experimental monitoring purposes, the system introduces a two-factor process to detect actions that should trigger warnings. These two factors are 1) detecting whether personnel have entered an alert zone and 2) detecting the occurrence of warning actions. When both conditions are met, the system initiates specific operations. In particular, if the system detects that personnel have entered the alert zone while performing warning actions, it immediately captures the relevant images and transmits them to a designated communication group. Simultaneously, these images are saved in an image management system for later review and tracking.

The research design and implementation process of this work are detailed in the Materials and Methods section, while the results of an application are presented in the Results section. In Section 4, we discuss the findings and analyze their implications. Finally, the Conclusion section summarizes the contributions of this study and outlines potential future research directions and opportunities for further development.

## 2 Materials and methods

**Fig. 1:**
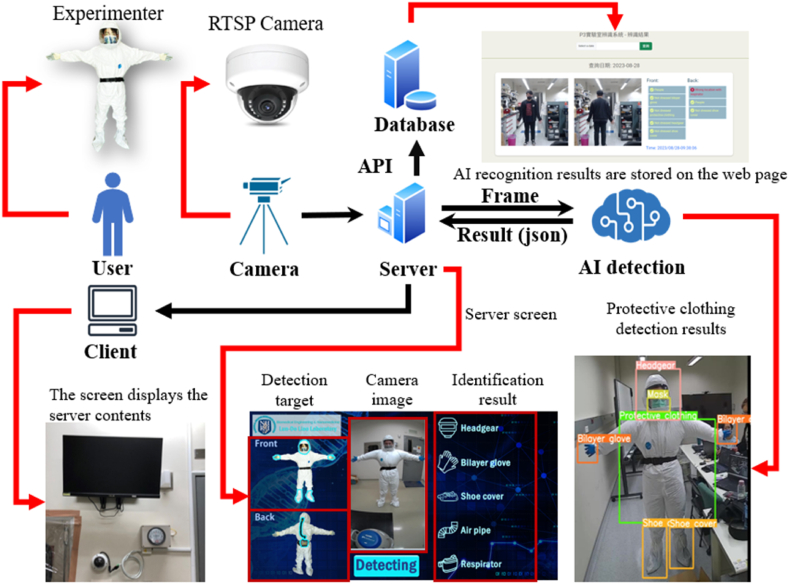
**The protective clothing recognition architecture employed in this study**. Once the experimental personnel finish dressing, they stand in front of the camera, allowing it to detect the human form and initiate the recognition process. The camera captures an image, which is then transmitted to the system. The AI model recognizes each piece of protective equipment worn and immediately displays its results on the screen to provide feedback to the experimental personnel. Simultaneously, the recognition results are stored in the backend database.

This study develops a BSL-3 laboratory management system consisting of two main components: 1) a protective clothing detection system for personnel entering the laboratory and 2) a monitoring system for personnel conducting experiments within the laboratory. The architecture of the protective clothing detection system, as illustrated in Fig. 1, employs You Only Look Once version 5s (YOLOv5s) as its base network model for the initial training process. A secondary model is then trained to assess whether personnel are properly wearing their protective clothing. The YOLOv5s model is integrated into the system to classify protective clothing into 15 categories: people, headgear, bilayer gloves, shoe covers, masks, protective suits, respirators, air pipes, the absence of shoe covers, incorrectly placed respirators, the absence of bilayer gloves, the absence of protective clothing, the absence of headgear, the absence of masks, and incorrectly placed air pipes.

**Fig. 2:**
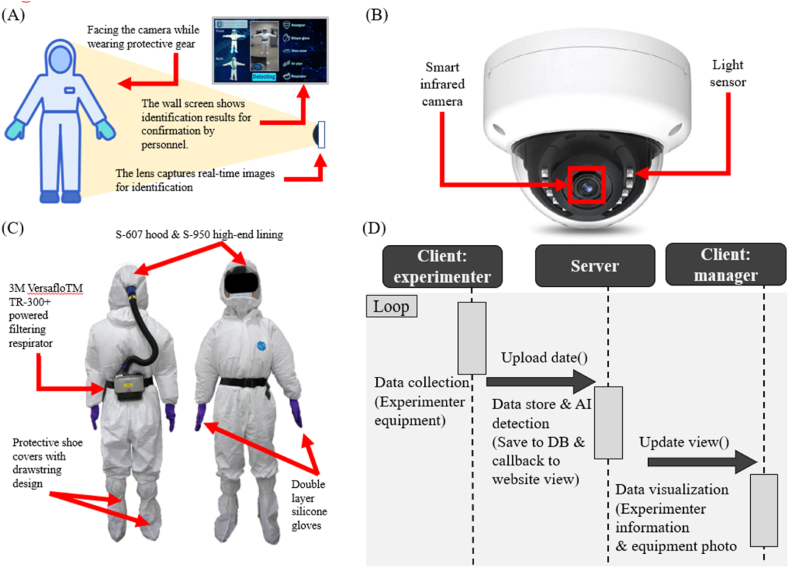
**Schematic diagrams related to BSL-3 laboratory safety. (A)** An illustration of experimental personnel facing the real-time recognition camera after dressing. They then follow the steps indicated on the screen to obtain results. **(B)** The semicircular network camera used in this study. **(C)** The equipment chosen by the experiment personnel, featuring the 3M VersafloTM TR-300+ powered air-purifying respirator. It consists of a hood, a breathing tube, and a main unit. The selected hood includes an S-607 hood, an S-950 premium inner shroud, double-layer silicone gloves, and protective shoe covers designed with drawstrings. **(D)** The cyclical workflow of the recognition process. Initially, data concerning the application of protective equipment by the user are acquired, and this step is followed by data storage and AI recognition. Finally, the recognition results are visually presented to the user.

The deep learning model is implemented via a web interface for AI detection, as shown in Fig. 2(A). After donning protective clothing, personnel stand in front of the camera for inspection purposes, where the system confirms the completeness of their protective gear. The recognition results are displayed on the screen. Traditionally, inspections rely on human visual assessments, but the integration of AI detection adds an extra layer of safety, reducing the risk of personnel being exposed to pathogens during experiments. The images captured during the AI detection process are stored on a server and uploaded to a cloud database along with the assessment results provided by the system. This enables future reviews of the entry and exit records to facilitate laboratory management. When an infection occurs, laboratory records can be accessed immediately to trace the health statuses of personnel based on their time logs.

In addition to protective clothing detection, the BSL-3 management system supports internal laboratory oversight. In this section, we describe the autonomous training process conducted by the warning action model, where YOLOv5s is employed as the foundational network. Three action categories are labeled: "none" (no warning action), "open" (opening a refrigerator containing pathogens), and "click" (pressing a button to centrifuge virus samples for research). The trained model is integrated into the developed monitoring system, which imports images taken by real-time streaming protocol (RTSP) cameras and defines alert zones to detect whether personnel have entered restricted areas. The detection results output by the AI model are then transmitted to the system.

We also plan to enhance the functionality of the image management system (Argo). Since the current system lacks data reception capabilities, we developed a proprietary port with an intranet transmission function. This port receives data from within the target laboratory and transmits it to the image management system in JSON format via HTTP POST. When personnel enter an alert zone and violate the set safety protocols, the system sends a warning message with the corresponding image to the designated line group. This system significantly enhances the ability to internally monitor personnel activities in a BSL-3 laboratory. After the camera captures an image, the system outputs both the associated alert zone status and the AI detection results. When both conditions are satisfied within the same frame, a warning message is automatically sent to the designated line group and reported to the server.

### 2.1 Semicircular web camera

This study utilizes real-time streaming images acquired from a hemispherical network camera (SR-C-A2-DF1-F3-IR; Spark, Taiwan) as inputs for the developed AI object recognition model. The camera lens is accessed via the Open Source Computer Vision Library (OpenCV) package in Python to capture RGB images (Fig. 2(B)). OpenCV, which was originally developed by Intel, is distributed under a Berkeley Software Distribution (BSD) license, which allows free use in both the commercial and research domains. It is one of the most comprehensive open-source tools for the development of computer vision methods, with applications in various fields such as facial recognition, gesture recognition, motion detection, object tracking, object recognition, and image segmentation. OpenCV supports several programming languages, including Java, Python, and C/C++, and is known for its high execution efficiency, making it ideal for use in real-time applications [14].

The first step involves adjusting the resolution of the captured RGB images to 1920 × 1080 via OpenCV. Since OpenCV reads images in a default blue–green–red (BGR) format, conversion to a red–green–blue (RGB) format is needed. One of the advantages of OpenCV is its ability to handle this conversion step with a single line of code. Once the conversion process is complete, the images are ready for the subsequent AI analysis stage.

### 2.2 Protective equipment

The current method for donning and doffing protective equipment follows the 2004 edition of the "BSL-3 Laboratory Simulated Education and Training" document issued by the National Institute of Infectious Diseases and Vaccinology at the National Health Research Institutes of Taiwan. This procedure aligns with the guidelines in the "Laboratory Biosafety Manual – Personal Protective Equipment" document of the World Health Organization (WHO); specifically, it is outlined in Chapter Six, which covers "Laboratory Coats, Gowns, Aprons, and Coveralls." This chapter provides detailed instructions on the correct sequence for donning and doffing multiple layers of personal protective equipment (PPE), with the degree of complexity varying depending on the type of PPE used [15]. In the 2021 edition of the Laboratory Biosafety Guidelines of Taiwan, additional regulations were added to specify the exact order in which laboratory personnel must don and doff their protective equipment. Adherence to these guidelines, including the strict sequence for applying and removing protective suits, is essential for preventing the accidental release of pathogens. Any deviation from these procedures may raise serious concerns about biosafety risks [16].

The protective equipment used in a BSL-3 laboratory includes inner and outer gloves, inner and outer shoe covers, a one-piece nonairtight chemical-resistant protective suit, a hood with a face shield, a respirator, and a mask, as shown in Fig. 2(C). Both the inner and outer gloves are made of latex. If any glove contamination is suspected during the experimental process, the outer gloves are immediately replaced [17]. The utilized protective suit is a DuPont Tyvek one-piece suit made of an antistatic material that offers protection against harmful dust and liquid splashes. The suit is noncytotoxic, nonallergenic, and nonirritating, making it widely used in the chemical, biomedical, and electronics manufacturing industries [18]. The respirator employed in the laboratory is the 3M Versaflo™ TR-300+ powered air-purifying respirator, which consists of a hood, a breathing tube, and a main unit. The hood includes an S-607 hood and an S-950 premium inner shroud. This portable powered respirator effectively filters out particulate matter, SO_2_, NOx, O_3_, and volatile organic compounds, which are common air pollutants [19]. This study focuses primarily on detecting whether personnel are wearing a complete set of protective equipment. To increase the efficiency and user-friendliness of the detection system, the system displays five critical equipment items that are frequently worn incorrectly or omitted: hoods, gloves, shoe covers, respirators, and tubing. While other categories are not displayed on the user interface (UI), detailed detection records are stored in the background of the system for reference when needed.

### 2.3 AI model training

The deep learning models that are commonly used for object recognition, such as convolutional neural networks (CNNs) [20], recurrent neural networks (RNNs) [21], U-Nets [22], and generative adversarial networks (GANs) [23], all have distinct architectures that are tailored to specific tasks. The YOLO series [[24], [25], [26]], for example, employs a single-shot forward propagation structure, where the entire input image passes through the neural network only once during forward propagation. This eliminates the need for multiple computations, resulting in faster processing speeds. Additionally, YOLO can simultaneously detect multiple objects belonging to different classes without requiring separate models or steps.

In this research, YOLOv5 is chosen for object detection because of its balance between speed and accuracy, making it highly suitable for the real-time detection of multiple targets in high-risk environments such as BSL-3 laboratories. YOLOv5 integrates several key features that enhance its performance, including a mosaic data augmentation module, an adaptive anchor calculation, a focus structure, a cross-stage partial (CSP) network, and the use of feature pyramid networks and path aggregation networks (FPNs + PANs). These features, combined with the generalized intersection-over-union (GIOU) loss function [27], optimize the detection process, enabling precise and efficient object recognition.

Mosaic data augmentation improves the generalizability of the model by combining four images into one during training, allowing the network to learn from diverse image contexts. This technique uses affine transformations to adjust the scale, rotation, and translation levels of the examined images, enhancing the ability of the model to detect objects under various conditions. YOLOv5 also utilizes an adaptive anchor calculation, which optimizes the dimensions of the anchor box by calculating the intersection over union (IoU) between the predicted and ground-truth boxes and refines the box sizes through gradient descent to achieve improved accuracy. The focus structure within YOLOv5 improves its feature extraction ability by downsampling the input images early in the network while preserving the key spatial information that they contain. This process involves convolutional layers and activation functions, specifically rectified linear unit (ReLU) functions, which enhance the ability of the network to filter out irrelevant details while maintaining essential features. The CSP network further improves the efficiency of the model by splitting the input feature map into two parts, allowing for a more effective gradient flow during backpropagation and reducing the incurred computational cost without sacrificing performance.

The integration of the FPN and PAN architectures by YOLOv5 enhances its multiscale feature fusion capabilities, ensuring that objects of various sizes are accurately detected across different layers of the network. The FPN aggregates features derived from different levels of the network, whereas the PAN strengthens the propagation of lower-level features to more precisely localize objects. These structures facilitate better gradient flows, improving both the detection and localization accuracies of the model. To optimize the bounding box predictions produced by the model, the GIOU loss function is used. The GIOU improves the traditional IoU by addressing edge cases in which the bounding boxes only partially overlap or fail to overlap entirely. By more effectively penalizing inaccurate predictions, the GIOU ensures that the model makes better localization decisions, particularly in complex detection scenarios.

In this study, YOLOv5 is trained on a dataset with a batch size of 32 at a resolution of 640x640 pixels, and the model undergoes 300 training iterations. The optimization process employs the adaptive moment estimation (Adam) optimizer, which uses backpropagation to adjust the model weights based on the gradient of the loss function. This configuration enables real-time detection to be conducted with high precision, satisfying the efficient and accurate monitoring requirements of this research for BSL-3 environments.

The labeling software used in this study is LabelImg [28], which directly generates annotation files in the format required by the YOLO series of networks. The hardware configuration employed in this research includes an Intel Core i7-13700H CPU, a 24-GB NVIDIA GeForce RTX 4090 GPU, and 32 GB of RAM, and the hardware runs on the Windows 10 Pro ×64 operating system.

Two models are trained in this study: 1) the protective clothing detection model and 2) the warning action model. The dataset used for the protective clothing detection model includes 2220 images annotated into 15 categories: people, headgear, bilayer gloves, shoe covers, masks, protective clothing, respirators, air pipes, missing shoe covers, incorrectly placed respirators, missing bilayer gloves, missing protective clothing, missing headgear, missing masks, and incorrectly placed air pipes. A total of 1776 images are used for training, while 444 images are set aside for validation purposes. The dataset employed for the warning action model comprises 3347 images labeled with 3 categories: “none”, “open”, and “click”. The "none" category contains 3683 occurrences, "open" possesses 1590 occurrences, and "click" includes 1704 occurrences. Among these images, 2678 are used for model training, with 669 reserved for validation.

### 2.4 Image processing and recognition system

This study uses OpenCV for image acquisition and stitching, whereas the recognition system utilizes the pose module contained in MediaPipe. When the system is initiated, OpenCV opens the camera and captures the observed scene. The image format is then converted from BGR to RGB, enabling MediaPipe to analyze the image. MediaPipe, which was developed by Google Research, is a multimedia machine learning framework that supports languages such as JavaScript, Python, and C++. It provides an API for functionalities such as 3D hand landmark tracking, BlazeFace facial detection, and object detection. The MediaPipe Pose model identifies 33 body landmarks, enabling recognition to be performed on the basis of these points. Once a complete human body is detected, a countdown timer starts. When the countdown reaches zero, the frame is stored on the server. An AI system then automatically assesses whether the protective equipment is being worn correctly. The operational workflow is illustrated in Fig. 2(D). After the processes of data collection, storage, and recognition are completed, the results are visualized and saved, and user feedback is provided.

### 2.5 The connection between the front-end web page and the backend server of the protective clothing identification system

The backend development framework chosen for this study is FastAPI, which is a high-performance web framework that was designed specifically for constructing application programming interface (API) services [29]. FastAPI supports Python versions 3.6 and above and is built on top of Pydantic and Starlette. The Pydantic library leverages Python-type hints to handle data validation, serialization, and documentation tasks (via a JSON schema). Starlette, which is a lightweight asynchronous server gateway interface (ASGI) framework, is well suited for building high-performance asynchronous services.

**Fig. 3:**
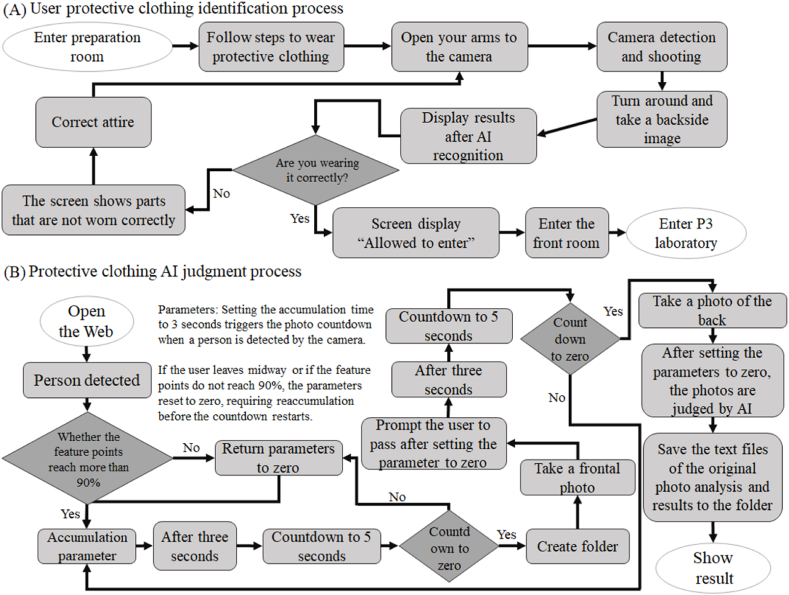
**Processes employed by the protective clothing inspection system for experimental personnel before entering a BSL-3 laboratory and the AI recognition module within this system. (A)** The process diagram of the protective clothing inspection system utilized for experimental personnel before entering the BSL-3 laboratory. After entering the preparation room, the experimental personnel follow the set dressing steps to don their protective equipment. When a person faces the camera with their arms outstretched, the camera detects this position, takes a photo, and prompts the person (through both text and voice) to turn around so that a back photo can be captured for identification purposes. After these steps are completed, the results are displayed on the screen. If incorrectly donned equipment is indicated, the personnel must correct this issue and undergo the identification process again. If the equipment is worn correctly, "Allowed to enter" is displayed, and the personnel can proceed to the anteroom to prepare for entry into the BSL-3 laboratory to perform experiments. **(B)** The AI recognition process performed by the protective clothing inspection system for experimental personnel before they may enter the BSL-3 laboratory. Upon opening the webpage, human detection begins; when 90 % of the human characteristic points are matched, the parameters accumulate for 3 s, initiating the countdown for beginning the recognition process. After 5 s, a front image is captured, and a folder is created for this detection task. The parameters are reset, and the user is prompted to turn around for another recognition countdown. After 5 s, their back image is captured and stored in the previously created folder. The AI judgment step is performed after resetting the parameters. After the analysis process, the original photos and analysis result text are stored in the folder displayed on the screen.

The system architecture is shown in Fig. 1, and the corresponding procedural flowchart is illustrated in Fig. 3(A). First, both the client and server sides of the web system are activated. The client side initiates the RGB camera to capture images, which are then transmitted to the server via the user datagram protocol (UDP). The UDP is selected due to its higher transmission speed than that of the transmission control protocol (TCP). While the UDP may experience data losses, the frame rate achieved for RGB images is approximately 30 frames per second (FPS), meaning that 30 frames are transmitted per second. The occasional loss of a few frames has a minimal effect on the overall performance, making the UDP a suitable choice. Once the server receives the images, the AI recognition system is triggered to perform the necessary AI assessments.

The AI judgment process is depicted in Fig. 3(B). When the camera detects a human figure, it first checks whether more than 90 % of the characteristic points of the body are identified for three consecutive seconds. This step ensures that an individual is properly positioned in front of the camera for detection. Once confirmed, the system initiates a countdown. After 5 s, the system captures an image to assess the protective clothing on the front side and prompts the individual to turn around. Following a 3-s pause, the system analyzes the protective clothing on the back side. Another countdown is initiated, and the system captures an image for back-side recognition. Finally, the results derived from both images are displayed on the screen and stored in the database.

The web application UI is built via HTML, CSS, and JavaScript. CSS is first used to define the styling properties of each UI element, such as its dimensions and font size. Then, HTML is employed to structure the layout and place the elements in their designated positions. Finally, JavaScript handles asynchronous updates, ensuring that the data produced by the AI recognition system are displayed in real time.

During the recognition process, researchers wearing protective clothing must follow the instructions provided by the system: turning toward the wall and then looking back at the screen to confirm the results. To enhance the degree of user convenience and improve the overall experience, English audio prompts such as "turn around" are added to signal the appropriate time at which the user should turn. These audio cues help prevent users from missing recognition prompts due to their unfamiliarity with the process or errors, minimizing the unnecessary expenditure of time and effort.

### 2.6 Internal image management system for a BSL-3 laboratory

**Fig. 4:**
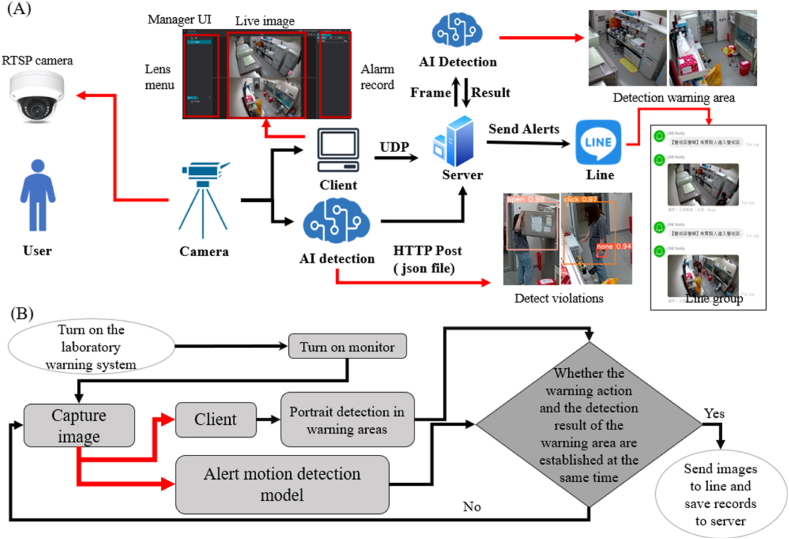
**Indoor monitoring schematic diagram and flowchart. (A)** A schematic diagram of the monitoring system employed for the experimental personnel during internal experiments. The camera installed in the control room is used to assess two factors (1: alert zone; 2: hand motion recognition). When both conditions are triggered, the server sends a warning message via a line group to alert the relevant personnel to take further action. **(B)** The flowchart of the monitoring system used for the experimental personnel during the internal experiments. The camera system continuously captures real-time images and assesses the two above factors (1: alert zone; 2: hand motion recognition). If both conditions are satisfied, an immediate warning message is sent, and the relevant information is stored on the server. If no recognition result is produced or only one condition is satisfied, the system continues to analyze the images at the next time node.

The Argo image management system serves as the foundation for the development of the proposed system, as shown in Fig. 4(A). The RTSP camera displays images on the client-side UI, where the system determines whether laboratory personnel are located within the alert zone and if any warning actions have been performed. If both conditions are met—indicating the presence of personnel in the alert zone and the execution of a warning action—the system notifies administrators via Line messaging. The first development step involves optimizing the UI to make the process of defining the alert zone boundaries more intuitive and user friendly. Next, the warning action model is integrated into the system (Fig. 4(B)), and an AI recognition system is established to evaluate two key factors: (1) whether laboratory personnel entering the BSL-3 laboratory are correctly wearing protective clothing and (2) whether personnel inside the laboratory are performing warning actions. When both conditions are triggered, i.e., when personnel enter the alert zone and perform a warning action, the system sends a notification to the administrators via Line, which is accompanied by an image. Detailed alert information is also stored in the system backend for future reference. These notifications, along with backend data records, provide real-time situational awareness and support management, ensuring the operational safety and efficiency of the BSL-3 laboratory. The design and integrated functionality of this system are expected to significantly improve laboratory management processes by providing enhanced overall performance and security.

## 3 Results

### 3.1 AI model training results obtained for the external protective clothing identification task conducted by the BSL-3 laboratory management system

**Fig. 5:**
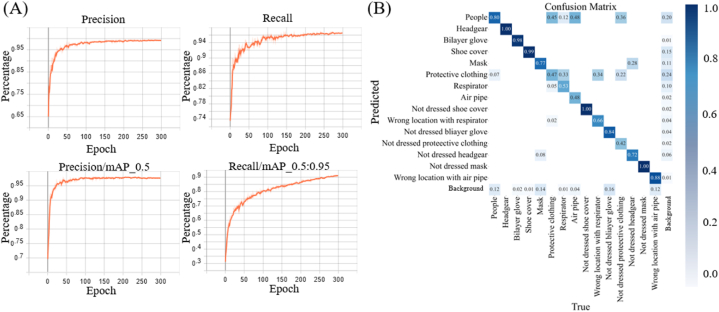
**Training results produced by the protective clothing detection model. (A)** The upper-left graph shows the accuracy curve obtained for each training iteration during the AI model training process of the protective clothing detection system before experimental personnel enter the BSL-3 laboratory. The upper-right graph displays the recall curve produced during each training iteration implemented by the same AI model. The lower-left graph illustrates the mAP values achieved with IoU thresholds greater than 0.5 during each iteration of the AI model training process. The lower-right graph shows the recall rate of the average mAP values achieved with IoU thresholds ranging from 0.5 to 0.95 for each training iteration of the AI model. **(B)** The confusion matrix used to validate the AI model contained in the protective clothing detection system for experimental personnel entering the BSL-3 laboratory.

The training dataset for the proposed system includes 15 labeled classes: people, headgear, bilayer gloves, shoe covers, masks, protective clothing, respirators, missing shoe covers, incorrectly placed respirators, missing bilayer gloves, missing protective clothing, missing headgear, missing masks, and incorrectly placed air pipes. A total of 2220 images are annotated, with 1776 used for model training and 444 employed for validation over 300 training iterations via the leave-one-out approach. The top-left graph in Fig. 5(A) shows the accuracy curve produced by the AI model during training. The accuracy surpasses 95 % after 50 iterations, improving gradually and ultimately reaching 97 %. The top-right graph in Fig. 5(A) shows the recall curve, with the recall rate reaching 94 % after 40 iterations and fluctuating slightly before stabilizing at 95 %.

The IoU threshold, which is a key metric for evaluating object detection and segmentation performance, measures the overlap between the predicted bounding box or segmentation mask and the actual target (ground truth). The bottom-left graph in Fig. 5(A) shows the mean average precision (mAP) values achieved for IoU thresholds greater than 0.5 during each training iteration. The mAP exceeds 95 % after 50 iterations and approaches 97 % during the later rounds. The bottom-right graph shows the recall of the average mAP values achieved for IoU thresholds ranging from 0.5 to 0.95. The recall reaches 70 % after 50 iterations, climbs to 90 % by the 280th iterations, and reaches 91 % after 300 iterations. These four graphs demonstrate that the model achieves a 95 % recall rate for its bounding box predictions, closely matches the actual targets, and has an overall detection accuracy of 94 %.

Fig. 5(B) presents the confusion matrix yielded by the model during the validation process. The matrix shows that the model accurately predicts all classes, demonstrating its strong ability to differentiate between various categories and confirming its robust classification capabilities. Based on the validation results, the model achieves 100 % accuracy for seven classes: "bilayer gloves," "shoe covers," "air pipes," "missing shoe covers," "incorrectly placed respirators," "missing bilayer gloves," "incorrectly placed air pipes," and "missing masks". Although some categories are misclassified as "background," the overall error rate remains low, averaging just 6.8 %. However, the "people" category produces a higher misclassification rate, with a 20 % chance of being incorrectly classified as "background." Additionally, the categories "protective clothing," "respirators," "air pipes," and "missing protective clothing" have an average misclassification probability of 35 %. Nevertheless, the use of the MediaPipe Pose model for conducting human body node recognition on the "people" category means that these errors do not significantly affect the overall workflow.

### 3.2 AI model training results obtained for the internal warning action recognition process implemented by the BSL-3 laboratory management system

**Fig. 6:**
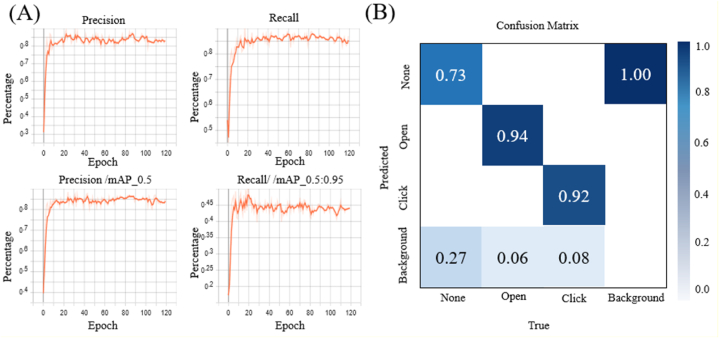
**Model training results produced for alert actions. (A)** The upper-left graph depicts the accuracy curve obtained during each iteration of the AI model training process conducted by the monitoring system for internal experiments. The upper-right graph illustrates the recall curve produced by the same AI model for each training iteration. The lower-left graph shows the mAP values achieved with IoU thresholds greater than 0.5 for each iteration during the AI model training process. The lower-right graph represents the recall rate of the mAP values attained with IoU thresholds ranging from 0.5 to 0.95 for each training iteration. **(B)** The confusion matrix representing the validation results yielded by the AI model within the internal experimental monitoring system.

The internal management system used in this study is depicted in Fig. 6. The label categories are divided into three classes: "none," "open," and "click." A total of 3347 images are annotated across these categories, with the "none" category appearing 3683 times, the "open" category appearing 1590 times, and the "click" category appearing 1704 times. Among these images, 2678 images are used for model training, while 669 images are set aside for validation. The model converges after 120 training iterations and is trained a total of 120 times. The data collection, labeling, and training processes of the AI neural network model are conducted via the leave-one-out approach. The upper-left graph in Fig. 6(A) shows the accuracy curve produced during each training iteration. The accuracy reaches 80 % after 10 iterations, fluctuates slightly, and ultimately reaches 83 %. The upper-right graph in Fig. 6(A) shows the recall curve, with the recall rate reaching 80 % after 10 iterations and increasing to 85 % by the end of the training procedure. The lower-left graph in Fig. 6(A) displays the average mAP achieved with an IoU threshold greater than 0.5 during each iteration. After 50 iterations, the mAP exceeds 95 % and gradually approaches 97 %. The lower-right graph shows the recall rate of the mAP values achieved with IoU thresholds ranging from 0.5 to 0.95 during each iteration. The recall reaches 70 % after 50 iterations, increases to 90 % by the 280th iteration, and reaches 91 % after 300 iterations. These four graphs demonstrate that the model achieves a 95 % recall rate for its bounding box predictions, closely approximating the actual targets. Additionally, the detection accuracy is highly reliable, reaching 94 %.

Fig. 6(B) shows the confusion matrix generated by the model on the validation set after completing the training process. This matrix is important because it evaluates the performance of the model by comparing its predictions with the actual categories contained in the validation set. When the predicted categories match the actual categories, the model is considered to have higher utility. According to the confusion matrix, the accuracies achieved for the two alert actions are 94 % and 92 %. However, the accuracy attained for the nonalert category is only 73 %, representing a higher likelihood of misclassifying nonalert actions as background. Nevertheless, the lower accuracy achieved for nonalert actions does not significantly impact the overall effectiveness of the model, as the primary focus concerns the recognition of alert actions.

### 3.3 Protective clothing monitoring system for BSL-3 laboratories

The target audience for the proposed system is laboratory personnel entering a BSL-3 laboratory. The functionality of the system includes capturing photos and conducting AI assessments to verify whether personnel are wearing complete protective gear. The collected data are stored on the server and can be accessed later for reference if needed.

Fig. 4(B) illustrates the first step of the system: initiating the camera. Once activated, the camera begins capturing images and detecting whether anyone is in front of the lens via MediaPipe Pose. When a person stands in front of the camera, MediaPipe Pose activates and connects 33 nodes to form a skeleton. The parameter "visibility" within MediaPipe Pose refers to the number of detected nodes out of 33, which helps the system determine whether the user is fully visible to the camera. If the visibility level exceeds a certain threshold, the system proceeds with the next stage of the program. Different poses influence the visibility value. For example, when the user's entire body is visible, the visibility reaches 0.989. However, if the user is too close to the camera, leading to partial body detection, the visibility may drop to 0.359. When the user turns around, the visibility level decreases slightly, reaching 0.881; this value is still higher than that produced when the user is too close to the camera. Based on these observations, the visibility threshold is set to 0.8.

Once the system is activated, detection begins. If a person is detected and the visibility level exceeds the set threshold, a countdown program is initiated. If the user leaves unexpectedly during the countdown, the program automatically exits to prevent the capture of incorrect images. When the countdown reaches zero, a folder named with the current timestamp is automatically created, and the captured frame is saved to this folder using OpenCV. The folder is linked to the server via FastAPI, allowing the images captured by the camera to be accessed by entering the correct path. This study aims to capture front and back images of users for analysis with YOLOv5. Therefore, the program is designed to proceed only when two images are present in the folder—one from the front and one from the back. Once both images are contained in the folder, the YOLOv5 analysis process is automatically initiated, and the results are stored in the same folder. By synchronizing with the access control times, the system can identify the user associated with a particular folder, facilitating future tracking.

Once the functionalities of the system are refined, the UI is designed with three main sections. The first section contains sample photos, allowing the experimenters to replicate the actions shown in the images to ensure accurate AI judgments. The second section displays real-time images so that the experimenters can see themselves and confirm that they are properly positioned within the camera frame. A countdown timer for capturing photos is shown above the real-time image. The third section presents the judgment results produced by the AI system. If an incorrect class is displayed or the confidence value falls below 0.45, the experimenters are prompted to recheck their protective equipment.

**Fig. 7:**
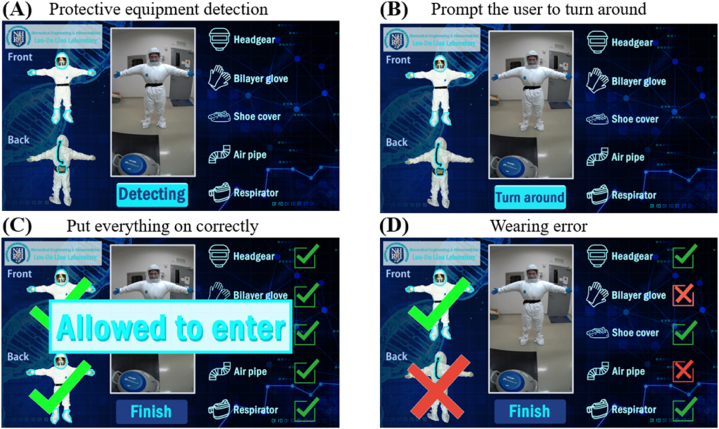
**Descriptions of the protective laboratory clothing detection interface. (A)** The actual operating interface of the web-based protective clothing detection system for personnel entering the BSL-3 laboratory. **(B)** After capturing the front-facing photo, visual and audio prompts are provided so that the user turns around; this is followed by a countdown implemented to prepare for capturing a back image. **(C)** After the photo capture step, the system displays the AI-determined results. If all categories are correct, the message "Allowed to enter" is displayed, indicating that the user is wearing the correct attire. **(D)** Categories that the AI system does not detect or misjudges. In such cases, the corresponding categories are marked with "X‴s. If personnel see this information, they know that they need to reinspect their attire.

The UI of the system is illustrated in Fig. 7(A). After the countdown finishes, a frontal photo is taken. The system then prompts the user with both visual and auditory cues to turn around for a back-side image, as shown in Fig. 7(B). Once the back photo is captured, the AI judgment results are displayed. If all categories are correctly identified, as shown in Fig. 7(C), the message "Allowed to enter" appears on the screen, indicating that the protective equipment is correctly worn by the personnel, permitting their entry into the BSL-3 laboratory. If the AI fails to recognize a category or detects improperly worn equipment, the result is displayed as shown in Fig. 7(D), with a cross marked on the corresponding category. When personnel see this, they are prompted to recheck their equipment. The decision threshold of the AI model is set at 0.45. If the postrecognition confidence value is less than 0.45 or improperly worn equipment is detected, the personnel are instructed to adjust their protective gear. For a demonstration of properly worn equipment, refer to Support Video 7(A), and for a demonstration of missing equipment, such as a breathing pipe or respirator, see Support Video 7(B). All judgment results are stored on the administrator's page, which is accessible only with the appropriate account permissions. In the future, administrators will be able to efficiently review personnel records directly from the administrator page, streamlining the BSL-3 laboratory management process.

**Fig. 8:**
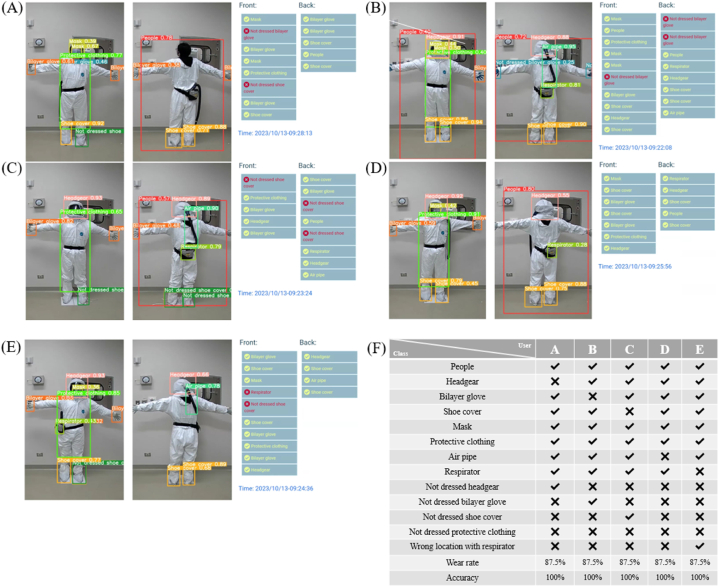
**Description of the background database used for protective laboratory clothing testing.** Common errors observed in terms of donning protective equipment: **(A)** headgear not worn, **(B)** gloves worn incorrectly, **(C)** shoelaces of shoe covers not fastened, **(D)** breathing tube not connected, and **(E)** respirator tilted. **(F)** Statistics of dressing categories (A)–(E).

Various validation scenarios are tested. Although 225 combinations of equipment categories are theoretically possible, only five common protective clothing oversights are identified under realistic conditions: missing headgear (Fig. 8(A)), incorrect glove usage (Fig. 8(B)), untied shoelaces on shoe covers (Fig. 8(C)), unconnected air pipes (Fig. 8(D)), and tilted respirators (Fig. 8(E)). These scenarios are summarized in Fig. 8(F). The backend of the system successfully recognizes all five situations, confirming the effectiveness of the method used in this study.

### 3.4 Laboratory control action monitoring system

**Fig. 9:**
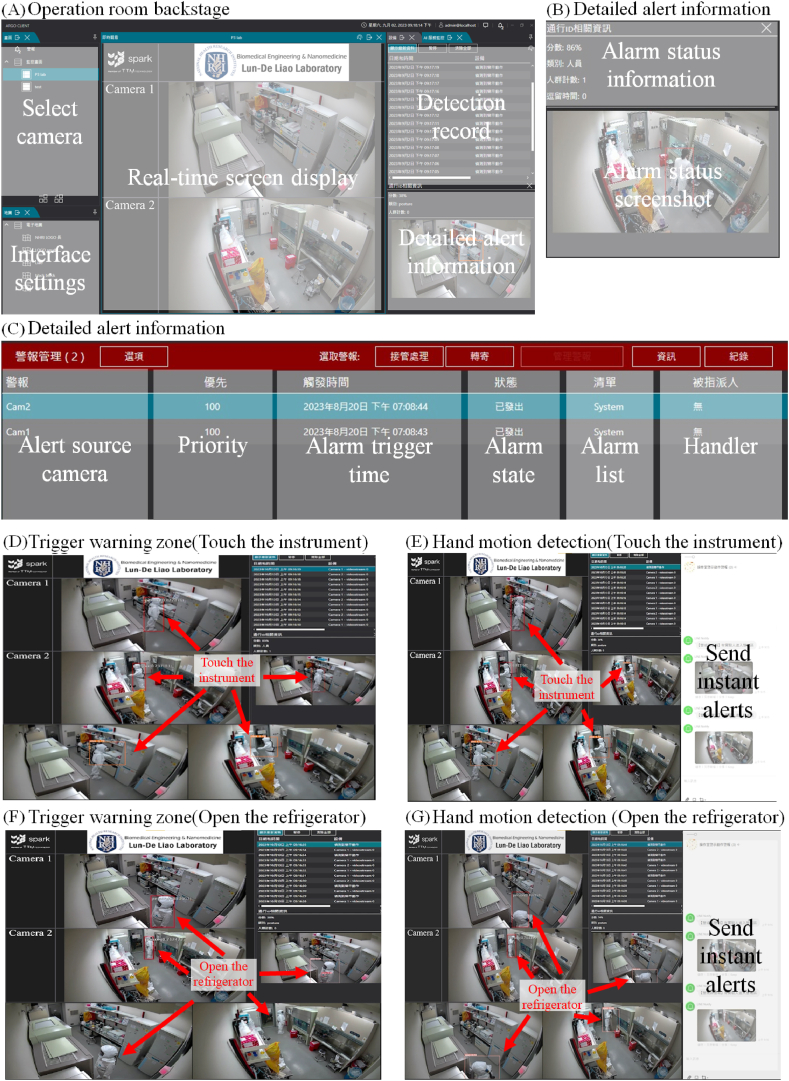
**Operating the indoor control action monitoring backend. (A)** The backend interface of the monitoring system employed during internal experiments, with the real-time image taken by the camera inside the operating room displayed at the center of the screen. **(B)** Clicking on the bottom right of the main screen to view further information about a specific record, such as its time, location, warning details, and corresponding image. **(C)** Clicking on the top right of the main screen to access details including the alarm source, the priority level, the trigger time, the alarm status, and information about the personnel in charge. **(D)** The centrifuge alert zone is triggered, but no centrifuge-related action is observed, so only the detection record is displayed at the top right. **(E)** The alert zone for using the centrifuge and hand movements are simultaneously detected, triggering line alert message transmission. **(F)** The refrigerator alert zone is triggered, but no action related to opening the refrigerator is observed, so only the detection record is displayed at the top right. **(G)** The alert zone for opening the refrigerator and hand movements are simultaneously detected, triggering line alert message transmission.

To enhance the overall user experience, we optimize the UI for the control room (Fig. 9), making it more intuitive and user friendly. This facilitates easier monitoring and management of the internal laboratory conditions by administrators. As shown in Fig. 9(A), the real-time image derived from the control room camera is displayed at the center of the screen. On the left side, a list of cameras is presented as clickable switches, allowing users to easily view multiple laboratories with various camera feeds. At the bottom right, a list of detected alert records is shown, enabling users to click on the entries to view additional details such as their time stamps, locations, warning descriptions, and corresponding images (Fig. 9(B)). In the upper right, records of the detected alerts and sent notifications are displayed (Fig. 9(C)), including information such as their alert sources, priority levels, trigger times, and alert statuses, as well as the details of the personnel responsible for managing the alerts. Fig. 9(D) and **(F)** show two alert zones detected without any interactions involving the centrifuge or refrigerator, resulting in records located in only the upper-right alert detection area. Fig. 9(E) and **(G)** show two alert zones that are detected simultaneously with hand movements involving the centrifuge and refrigerator opening. When both conditions are satisfied, Line alert messages are triggered and transmitted.

For further demonstrations, refer to Support **Video 9(A)** for the detection of the alert zone in front of the centrifuge; Support Video 9(B) for the simultaneous detection of the alert zone and hand movements involving the centrifuge, thus triggering line alert messages; Support Video 9(C) for the detection of the alert zone in front of the refrigerator; and Support Video 9(D) for the simultaneous detection of the alert zone and hand movements involving the refrigerator, which also triggers line alert messages.

This system utilizes a dual-factor assessment scheme, which is activated when laboratory personnel are detected within an alert zone and when violating actions are identified. Once both conditions are satisfied, the system sends a Line alert message along with the current image. Expanding these functionalities will enhance the BSL-3 laboratory management system, improving the efficiency and security achieved when monitoring and managing internal laboratory activities.

## 4 Discussion

The BSL system originated in the early 1970s, when it was proposed by the CDC in the United States to mitigate the risks of laboratory personnel contracting infectious diseases during viral experiments. The system is divided into four levels, BSL-1 through BSL-4, each of which corresponds to a different risk level and type of research involving infectious biological materials. BSL-3 laboratories are designated high-risk environments and are used to study moderately infectious biological agents. These standards are referenced globally to protect both laboratory personnel and the environment from biological hazards (see Table 1). In laboratory management scenarios, external auditing mechanisms are essential for identifying potential biosafety risks.

In a BSL-3 laboratory, the donning sequence is crucial for minimizing the risk of personnel coming into contact with highly infectious pathogens. After donning the inner protective equipment, personnel enter the anteroom to put on their respirators and face masks. Photographs are then taken, and AI recognition is used to verify that the protective gear is properly worn and that safety standards are met. If the AI results confirm compliance, the personnel are allowed to enter the main laboratory (see Fig. 2(A)). Emphasizing the correct donning sequence ensures that all protective equipment is worn properly, reducing potential risks and safeguarding the safety of laboratory personnel.

The system can be automatically initiated by opening a web browser. During the laboratory entry process, the existing procedures are enhanced by integrating the recognition capabilities of the developed system. This study introduces an additional confirmation step for laboratory personnel, ensuring a meticulous entry process that minimizes the risk of infection during experiments. The system also stores the data generated during this process on the server for future reference by administrators. By incorporating personnel photos and AI recognition results, the proposed system is expected to significantly improve the efficiency of BSL-3 laboratory management tasks.

The AI model developed for the BSL-3 protective laboratory clothing management system in this study achieves a final precision rate of 95 % and a recall of 94 %. The confusion matrix constructed for validation further confirms the accuracy of the model. According to the validation results, 100 % accuracy is achieved for the headgear, missing shoe cover, and missing mask classes. When applying this model on the web, both front and back images of the user must be captured to implement an AI assessment. As a result, the program is configured to proceed only when two camera images are saved in the designated folder. Once these two images are detected, the AI assessment process automatically begins, and the results are stored in the same folder (Fig. 3(B)). By synchronizing the folder with the timestamp of the access control system, the system can identify the user based on their images, facilitating future tracking (Fig. 8(F)).

Incorrect donning sequences lead to visible appearance differences. The proposed model can detect specific donning sequence errors, such as the incorrectly equipped gloves caused by not following the dressing order (refer to Support Video 10). The correct sequence involves donning the first layer of gloves, cutting a small opening in the protective suit for the thumb to pass through, and then putting on the second layer of gloves (Support video 10(A)). Wearing one layer of gloves first and immediately donning the second layer without cutting an opening in the protective suit for the thumb is an incorrect donning sequence (Support Video 10(B)).

In the conventional method, two laboratory personnel verify each other's protective clothing, which takes an average of 30 s per person. In contrast, the method developed in this study uses automatic detection to identify all equipment, completing the process in just 10 s—a threefold speed improvement. Additionally, this approach reduces the effort required from laboratory personnel, allowing them to focus more on their research activities.

A dual-factor alert system is integrated for internally managing BSL-3 laboratories. The self-trained alert action model achieves a precision rate of 83 % and a recall of 85 %. According to the confusion matrix constructed for verification purposes, the accuracies attained by the model in terms of recognizing the two alert actions reach 94 % and 92 %, confirming the identification accuracy of this approach. The modelling results are transmitted to the internal management system of the laboratory in JSON format via HTTP Post. This system can determine whether personnel are located in the alert zone, and if both conditions are satisfied, a warning message is sent to the Line app (see Fig. 4(B)), enabling more efficient BSL-3 laboratory management by administrative personnel.

In the current management approach, investigations are typically conducted only after an incident occurs; this strategy requires significant manpower to review surveillance footage over extended periods to identify the root cause of the target incident. In some cases, footage may be overwritten, resulting in a lack of backup data and the inability to determine the origin of the problem. The internal management system developed in this study integrates a dual-factor alert system to increase its accuracy. When an alert is detected, administrators are immediately notified, and the relevant information is recorded in the database (Fig. 9(B) and (C)). This enables administrators to respond promptly and access the necessary information in real time, effectively preventing injuries and avoiding issues caused by missing backups.

In the literature on BSL-3 laboratory management, no information concerning the use of AI for managing such facilities is available. This study integrates AI into BSL-3 laboratory management to reduce the imposed manpower burden, increase data integrity, shorten query times, and minimize human errors. This innovative approach represents the first research conducted worldwide on the application of AI in BSL-3 laboratory management scenarios. Future plans include refining the system by incorporating detection and recognition methods for large laboratory equipment and adding an extra layer of safety for instrument use cases. Additionally, since laboratory personnel undergo training to ensure safe practices, a training process-assisted recognition system is being developed to further reduce the pathogen exposure risks faced by laboratory staff.

## 5 Conclusion

This study focuses on researching viruses in a BSL-3 laboratory, including the development of standard operating procedures (SOPs) and a monitoring system that is aligned with the safety protocols implemented by from the National Institute of Infectious Diseases and the Health Promotion Administration of Taiwan. The proposed BSL-3 management system integrates both external protective suit monitoring and internal laboratory oversight.

To conduct external monitoring via the web, AI is used to automatically recognize protective suits, achieving 97.52 % precision and 97.03 % recall. The verification results obtained for protective clothing detection cover seven categories: bilayer gloves, shoe covers, air pipes, missing shoe covers, incorrectly placed respirators, missing bilayer gloves, incorrectly placed air pipes, and missing masks. The system achieves 100 % accuracy in all categories. Although some categories are not detected or misclassified as background information, the average error rate is only 6.8 %. This method also increases the speed of recognition by 300 %. The internal laboratory monitoring strategy, which uses YOLOv5s, detects alert actions with 90 % precision and 85 % recall. According to the confusion matrix constructed to verify indoor control actions, the accuracies achieved for the two warning actions reach 94 % and 92 %, confirming the accuracy of the model. The system also triggers Line notifications for administrators, improving its operational efficiency. This real-time approach enhances the degree of protection provided to laboratory personnel.

The versatility of the proposed AI system extends beyond BSL-3 laboratories, and it can be applied in various fields, including factory assembly line management [13,30,31]. The main goal of this research is to safeguard frontline operators from infection risks in hazardous environments, with prompt system implementation set as the priority to improve the protection provided for laboratory personnel.

This research represents a significant advancement in laboratory safety by utilizing AI to automate critical safety checks, reducing human errors and increasing efficiency. The real-time alerts and monitoring capabilities of the system enable swift responses to potential hazards, providing a scalable and effective solution for protecting personnel in high-risk environments. Although this study successfully develops and implements an AI-based monitoring system for achieved PPE compliance in BSL-3 laboratories, an ablation study—which can be performed to evaluate the contributions of different model components—is not conducted. This decision was made because the model architecture was not modified. However, we recognize the value of such an analysis for understanding how each component impacts the overall performance of a system. In future work, we plan to incorporate an ablation study to further refine the proposed model and gain deeper insights into its functionality. This will allow us to assess the significance of each component contained in the monitoring system and enhance the accuracy and reliability of the system.

While the current system demonstrates strong performance, future work will focus on addressing its minor detection errors and further refining the AI models to improve their accuracy. An ablation study may also be conducted to evaluate the contribution of each system component. Additionally, the possibility of expanding the applications of the system to other safety-critical environments, such as chemical laboratories and biomanufacturing facilities, will be explored. Furthermore, incorporating predictive analytics could enable the system to foresee potential hazards, enhancing the proactive safety management task in high-risk laboratories. This research lays the groundwork for developing advanced AI-driven safety systems that can protect frontline workers in various hazardous environments.

Disclosures/conflicts of interest.

The authors declare that there are no conflicts of interest.

## CRediT authorship contribution statement

**Yi-Ling Fan:** Writing – original draft, Visualization, Investigation, Funding acquisition, Formal analysis, Data curation, Conceptualization. **Ching-Han Hsu:** Writing – original draft, Project administration, Conceptualization. **Ju-Yu Wu:** Data curation, Conceptualization. **Ying-Ying Tsai:** Visualization, Validation, Data curation. **Wei J. Chen:** Supervision. **Min-Shi Lee:** Supervision. **Fang-Rong Hsu:** Formal analysis, Data curation, Conceptualization. **Lun-De Liao:** Writing – review & editing, Writing – original draft, Visualization, Validation, Supervision, Project administration.

## Data availability statement

The data that support the findings of this study are available from the corresponding author upon reasonable request.

## Declaration of competing interest

The authors declare the following financial interests/personal relationships which may be considered as potential competing interests: Lun-De Liao reports was provided by National Health Research Institutes. Lun-De Liao reports a relationship with National Health Research Institutes that includes:. Lun-De Liao has patent pending to Licensee. If there are other authors, they declare that they have no known competing financial interests or personal relationships that could have appeared to influence the work reported in this paper.

## References

[1] Ta L., Gosa L., Nathanson D.A., Yong W.H. (2019). Biobanking: Methods and Protocols.

[2] J. Y. Richmond, "The 1, 2, 3's of Biosafety Levels." Centers for Disease Control and Prevention.

[3] Wang K., Zhu X., Xu J. (2020/06/01 2020). Laboratory biosafety Considerations of SARS-CoV-2 at biosafety level 2. Health Security.

[4] Zaki A.N. (Nov 2010). Biosafety and biosecurity measures: management of biosafety level 3 facilities. Int. J. Antimicrob. Agents.

[5] Le Duc J.W. (2008). Framework for Leadership and training of biosafety level 4 laboratory workers. Emerging Infectious Disease journal.

[6] Wurtz N. (2016). Survey of laboratory-acquired infections around the world in biosafety level 3 and 4 laboratories. Eur. J. Clin. Microbiol. Infect. Dis..

[7] Shannon G.W., Willoughby J. (2004). Severe acute respiratory syndrome (SARS) in Asia: a medical geographic perspective. Eurasian Geogr. Econ..

[8] Li L. (2005). Biosafety level 3 laboratory for Autopsies of Patients with severe acute respiratory syndrome: Principles, practices, and Prospects. Clin. Infect. Dis..

[9] Bang E., Oh S., Chang H.E., Shin I.S., Park K.U., Kim E.S. (2022). Zika virus infection during research vaccine development: investigation of the laboratory-acquired infection via Nanopore Whole-Genome sequencing. Front. Cell. Infect. Microbiol..

[10] S H.D.U., Meechan J.P. Paul J. (2020). Biosafety in Microbiological and Biomedical Laboratories.

[11] Wang X., Niu D., Luo P., Zhu C., Ding L., Huang K. (6-8 Nov. 2020 2020). 2020 Chinese Automation Congress (CAC).

[12] Wu J.Y., Ching C.T., Wang H.D., Liao L.D. (Nov 30 2022). Emerging wearable Biosensor Technologies for stress monitoring and their real-world applications. Biosensors.

[13] Ku C.J., Wang Y., Chang C.Y., Wu M.T., Dai S.T., Liao L.D. (Nov 2022). Noninvasive blood oxygen, heartbeat rate, and blood pressure parameter monitoring by photoplethysmography signals. Heliyon.

[14] Bradski G. (2000). The openCV library. Dr. Dobb's J. Softw. Tools Prof. Program..

[15] (2004). Laboratory Biosafety Manual.

[16] Holmes K.L. (2014). International Society for the Advancement of Cytometry cell sorter biosafety standards. Cytometry.

[17] Keten D., Okdemir E., Keten A. (2020). Precautions in postmortem examinations in Covid-19-Related deaths: Recommendations from Germany. Journal of forensic and legal medicine.

[18] Wagner A., Dorawa P. (2016). Research on biophysical properties of protective clothing. Autex Res. J..

[19] Aranda A., Díaz-de-Mera Y., Jarama I. (2018). Could portable powered respirators help us avoid the exposure to air pollution?. Air Quality, Atmosphere & Health.

[20] Lecun Y., Bottou L., Bengio Y., Haffner P. (1998). Gradient-based learning applied to document recognition. Proc. IEEE.

[21] Rumelhart D.E., Hinton G.E., Williams R.J. (1986). Learning representations by back-propagating errors. Nature.

[22] Ronneberger O., Fischer P., Brox T., Navab N., Hornegger J., Wells W.M., Frangi A.F. (2015//2015). Medical Image Computing and Computer-Assisted Intervention – MICCAI 2015.

[23] Goodfellow I.P.-A., Mirza Jean, Mehdi, Xu Bing, Warde-Farley David, Ozair Sherjil, Courville Aaron, Bengio Yoshua (2014).

[24] Redmon J., Divvala S., Girshick R., Farhadi A. (2016). 2016 IEEE Conference on Computer Vision and Pattern Recognition (CVPR).

[25] Redmon J., Farhadi A. (2017). 2017 IEEE Conference on Computer Vision and Pattern Recognition (CVPR).

[26] Wang C.Y., Bochkovskiy A., Liao H.Y.M. (2021). 2021 IEEE/CVF Conference on Computer Vision and Pattern Recognition (CVPR).

[27] Zhu X., Lyu S., Wang X., Zhao Q. (2021). Proceedings of the IEEE/CVF International Conference on Computer Vision.

[28] Tzutalin (2017). "Labelimg,". https://github.com/HumanSignal/labelImg.

[29] Bansal P., Ouda A. (2022). 2022 International Symposium on Networks, Computers and Communications (ISNCC).

[30] Liu W.L. (2023). An IoT-based smart mosquito trap system embedded with real-time mosquito image processing by neural networks for mosquito surveillance. Front. Bioeng. Biotechnol..

[31] Wu J.Y., Wang Y., Ching C.T.S., Wang H.D., Liao L.D. (2023). IoT-based wearable health monitoring device and its validation for potential critical and emergency applications. Front. Public Health.

